# Frequent Arousal from Hibernation Linked to Severity of Infection and Mortality in Bats with White-Nose Syndrome

**DOI:** 10.1371/journal.pone.0038920

**Published:** 2012-06-20

**Authors:** DeeAnn M. Reeder, Craig L. Frank, Gregory G. Turner, Carol U. Meteyer, Allen Kurta, Eric R. Britzke, Megan E. Vodzak, Scott R. Darling, Craig W. Stihler, Alan C. Hicks, Roymon Jacob, Laura E. Grieneisen, Sarah A. Brownlee, Laura K. Muller, David S. Blehert

**Affiliations:** 1 Department of Biology, Bucknell University, Lewisburg, Pennsylvania, United States of America; 2 Department of Biological Sciences, Fordham University, Armonk, New York, United States of America; 3 Pennsylvania Game Commission, Harrisburg, Pennsylvania, United States of America; 4 U.S. Geological Survey–National Wildlife Health Center, Madison, Wisconsin, United States of America; 5 Department of Biology, Eastern Michigan University, Ypsilanti, Michigan, United States of America; 6 U.S. Army Engineer Research and Development Center, Vicksburg, Mississippi, United States of America; 7 Vermont Fish and Wildlife Department, Rutland, Vermont, United States of America; 8 West Virginia Division of Natural Resources, Elkins, West Virginia, United States of America; 9 New York State Department of Environmental Conservation, Albany, New York, United States of America; University of Bern, Switzerland

## Abstract

White-nose syndrome (WNS), an emerging infectious disease that has killed over 5.5 million hibernating bats, is named for the causative agent, a white fungus (*Geomyces destructans* (Gd)) that invades the skin of torpid bats. During hibernation, arousals to warm (euthermic) body temperatures are normal but deplete fat stores. Temperature-sensitive dataloggers were attached to the backs of 504 free-ranging little brown bats (*Myotis lucifugus*) in hibernacula located throughout the northeastern USA. Dataloggers were retrieved at the end of the hibernation season and complete profiles of skin temperature data were available from 83 bats, which were categorized as: (1) unaffected, (2) WNS-affected but alive at time of datalogger removal, or (3) WNS-affected but found dead at time of datalogger removal. Histological confirmation of WNS severity (as indexed by degree of fungal infection) as well as confirmation of presence/absence of DNA from Gd by PCR was determined for 26 animals. We demonstrated that WNS-affected bats aroused to euthermic body temperatures more frequently than unaffected bats, likely contributing to subsequent mortality. Within the subset of WNS-affected bats that were found dead at the time of datalogger removal, the number of arousal bouts since datalogger attachment significantly predicted date of death. Additionally, the severity of cutaneous Gd infection correlated with the number of arousal episodes from torpor during hibernation. Thus, increased frequency of arousal from torpor likely contributes to WNS-associated mortality, but the question of how Gd infection induces increased arousals remains unanswered.

## Introduction

**Figure 1:**
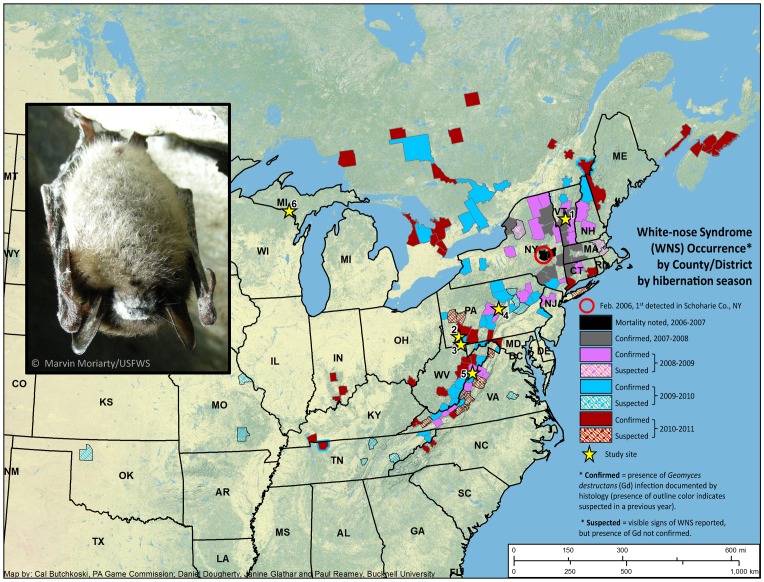
Distribution and spread of WNS throughout North America. Spread of WNS by hibernation season through the winter of 2010–2011 is shown along with locations of study sites, indicated by stars (see also Table 1). Confirmed sites have been officially reported by each state or province based upon histological confirmation of infection with the fungal pathogen *Geomyces destructans* (Gd); bats from suspect sites have clinical signs of WNS but lack laboratory confirmation. The inset shows a little brown bat infected with Gd from site #1 in Vermont. This site was WNS confirmed in 2008–2009, when bats were studied. Bats from site # 2 in Pennsylvania were studied in 2008–2009 (for 8 weeks only in the spring), when no signs of WNS were present, in 2009–2010, when a single bat from this site showed infection with Gd without mass mortality and in 2010–2011, when bats in this site were heavily infected. Bats from site #3 in Pennsylvania were studied in 2008–2009 (no WNS), 2009–2010 (when Gd was noted but without mass mortality) and in 2010–2011, when bats in this site were heavily infected. Bats from site #4 in Pennsylvania were studied in 2009–2010 (for 8 weeks only in the spring), when bats were heavily infected. Bats from site #5 in West Virginia were studied in 2008–2009, when there was no evidence of Gd presence – which was also the case for bats from site #6 in Michigan, which were studied all three years.

White-nose syndrome (WNS) is estimated to be responsible for the deaths of at least 5.7 to 6.7 million hibernating bats in the eastern United States and Canada [1], [2]. Clinical signs of WNS were first observed at a single cave in New York State during the winter of 2006–2007 and as of April 2012, WNS has spread to over 200 hibernacula in 19 U.S. states and four Canadian provinces (Fig. 1 [2], [3]). Bats with WNS display a number of aberrant behaviors, and in many instances they have depleted fat stores. Thus far, WNS affects at least six (and possibly nine) species of hibernating insectivorous bats [2], including some classified as endangered or threatened. The little brown bat (or, little brown myotis, *Myotis lucifugus*), which was once the most common hibernating bat in the American Northeast (NE), has incurred an average of 91% mortality in sites that have been affected for at least two years [2] and mathematical models indicate that this species may go extinct in the NE within 16 years [4]. A white fungus identified as *Geomyces destructans* (Gd) grows on the muzzle, wings, and ears of bats suffering from WNS starting in late January/early February [3], [5], [6]. Recent laboratory experiments have demonstrated that cutaneous infection with this fungus is the cause of WNS, but it is not fully understood how such an infection produces mortality during hibernation [7]. It is hypothesized that infection by Gd disrupts normal physiological functions, such as water balance [8] or other aspects of hibernation physiology, including use of torpor [9].

For insectivorous bats that live in northern temperate zones, such as those affected by WNS, food is primarily available from late spring to early autumn and absent during winter. Bats survive this winter energetic bottleneck by building stores of body fat (depot fat) in late summer and early autumn and by conserving metabolic energy through hibernation. In little brown bats, body fat increases from approximately 7% of total mass (∼6 g) during summer to 27% of total mass (∼9 g) prior to hibernation, an increase of 3 g or more in body mass [10], [11]. This depot fat is the sole energy source during the hibernating period, when body temperature (T_b_) and metabolic rate are both greatly reduced. Because their energetic costs in the subsequent spring are greater than those of males, female little brown bats enter hibernation with higher body mass indexes (BMI) and manage their energy stores during hibernation more efficiently than males [12]. Minimum metabolic rates during mammalian torpor can be <5% of basal metabolic rate with T_b_ close to ambient temperature (2° to 8° for bats) [13], [14]. However, hibernators do not remain torpid throughout hibernation; instead bouts of torpor last from days to weeks, interrupted by brief arousal episodes involving periods of high metabolic rate and euthermic T_b_ [15]. Earlier studies demonstrated that healthy, free-ranging little brown bats hibernating at ambient temperatures of 5–6°C have torpor bouts lasting between 12.4 and 19.7 days [16], [17], with arousal episodes lasting 1–2 hours.

**Table 1:** Temperature-sensitive datalogger deployment and retrieval (bat recapture) data, by study year, hibernacula site number (see Fig. 1), and sex for the 504 loggers deployed.

Site#	2008–2009				2009–2010				2010–2011			
	deployed*	retrieved*	down-loaded	included in final analyses**	deployed*	retrieved*	down-loaded	included in final analyses**	deployed*	retrieved*	down-loaded	included in final analyses**
1 (VT)	16/14 [11/6/08]	6/7 [3/17/09]	6/7	5/7								
2 (PA)	20/19+[1/27/09]	13/13 [3/24/09]	7/7	3/3	41/41 [11/13/09]	25/26 [3/25/10]	13/8	13/8	22/18 [11/18/10]	4/4 [3/10/11]	3/1	3/1
3 (PA)	15/15 [11/3/08]	9/4 [3/23/09]	8/2	8/2	40/30 [11/12/09]	9/8 [3/17/10]	7/2	7/2	22/7 [11/19/10]	8/1 [3/2/11]	7/1	7/1
4 (PA)					35/25+[1/6/10]	7/1 [3/11/10]	7/0	4/0				
5 (WV)	21/21+[1/29/09]	7/7 [3/23/09]	2/4	2/4								
6 (MI)	15/15 [11/7/08]	7/9 [3/21/09]	7/5	7/5	13/13 [11/14/09]	9/6 [3/27/10]	4/2	4/2	14/12 [11/6/10]	10/8 [3/26/11]	0/1	0/1

Whether data were successfully downloaded from the logger and ultimately used in the analyses of this paper, are also described.

* # of males/# of females and date of deployment or retrieval of loggers.

** bats were occasionally excluded from analyses due to incomplete data (e.g., BMI not recorded) or problems with downloaded data.

+ loggers only deployed mid-winter (January-March) as opposed to the full-hibernation season (November-March).

Although euthermic periods account for approximately 1% of the total time budget during winter, about 80–90% of the energy (depot fat) used during hibernation is consumed during these periodic arousals from torpor, because metabolic rate greatly increases with increased T_b_ [13], [18]. The amount of depot fat expended during each arousal episode (not including flight) for hibernating little brown bats is about 107.9 mg [18]. While the function of arousal episodes in hibernators is poorly understood and likely multifactorial [19], the fact that every mammalian hibernator periodically arouses from torpor at great energetic cost indicates the benefits must be significant.

We tested the hypothesis that WNS reduces the length of torpor bouts during hibernation in free-ranging little brown bats. We predicted that a primary cause of the increased mortality/disease state associated with WNS is abnormally shortened torpor bouts, due to more frequent arousal episodes, as was shown previously for one affected free-ranging bat in late hibernation [20] and recently for a group of experimentally infected bats held in captivity [21]. We also predicted that greater body fat stores at the beginning of hibernation, as estimated by BMI, would mediate the negative effects of frequent arousals. These predictions were tested in field studies on free-ranging little brown bats conducted at multiple sites (Fig. 1) over three hibernation seasons. Skin temperature (T_sk_), which correlates well with T_b_ in small insectivorous bats, and which has been used extensively to study mammalian hibernation [22], was measured with temperature-sensitive dataloggers attached to the backs of WNS-affected and unaffected bats. Hibernation patterns in relation to the stage of infection by Gd were also analyzed for a small sample of bats for which data were available on fungal presence (PCR) and degree of infection (histopathology).

## Materials and Methods

### Permits and Permissions

This study was carried out in strict accordance with the recommendations in the Guide for the Care and Use of Laboratory Animals of the National Institutes of Health. The protocol was approved by the Institutional Animal Care and Use Committee at Bucknell University (protocol number DMR-02). In the states of VT and WV, research was conducted by state wildlife officials (SRD with Vermont Fish and Wildlife Department and CWS with WV Department of Natural Resources) on non-endangered bats; thus numbered permits were not required or issued. In Michigan, research was conducted each year under MI Scientific Collector’s Permit SC620 from the Michigan Department of Natural Resources to AK. In PA, research was conducted each year under PA Game Commission permits to DMR (84-2008; 70-2009; 183-2010), in collaboration with GGT, a wildlife biologist for the state of PA. In accordance with the permits and with state wildlife policies, research was either conducted on state land or on private property, with the explicit permission of private landowners.

### Temperature Tracking

Temperature-sensitive dataloggers were programmed to read skin temperature (T_sk_) every 30 min and were attached to 504 bats over the course of three winters at six different hibernacula using standard methods [22]. Temperature readings could not be collected more frequently due to constraints on datalogger memory and the need to record continuous data for up to five months. To maximize recapture rates, bats with loggers were recaptured in March of each year, several weeks prior to the ‘normal’ time of emergence from hibernation. Loggers weighted about 1.1 g and were either purchased commercially (iBBat or WeeTagLites, AlphaMach, Inc., British Columbia, Canada) or were constructed by the authors (DMR and GGT). Appendix S1 describes and illustrates the methods for making these dataloggers from Thermochron DS1922L iButtons (Maxim Integrated Products, Inc., California, USA), modified from the techniques of Lovegrove [23]. Table 1 provides a summary of loggers deployed, retrieved, and downloaded successfully, by site, year, and sex.

Study sites were widely distributed and located in Vermont, West Virginia, Pennsylvania, and the Upper Peninsula of Michigan (Fig. 1). Among loggers retrieved, success rates varied. WeeTagLites failed at a rate of up to 90% whereas loggers constructed by the authors failed about 20% of the time. Overall 111 of 190 loggers retrieved yielded usable data, an average of 58.4%. We expected to recover less than half the loggers placed in the field and expected datalogger failure as well, which is why so many loggers were deployed. Of the 190 bats from which loggers were retrieved, 17 were found dead (four of which were in suitable post-mortem condition to perform histology analysis). For the 173 live bats recaptured in the spring, loggers were removed, and the animal was either released (N = 126) or euthanized for measurement of immune function and other physiological parameters for a separate study (N = 25) or for histology analysis (N = 22), as described below.

### PCR and Histology

Wing skin samples (approximately 3 mm X 3 mm each) were collected from a subset of freshly euthanized animals (N = 26). Nucleic acid was extracted from each skin sample using the Gentra Puregene genomic DNA purification kit (Qiagen Inc., Valencia, CA) per the manufacturer’s instructions (solid tissues protocol), with the following modifications: proteinase K was added to a final concentration of 0.5 mg/ml during the cell lysis procedure and no RNase treatment was performed. To determine presence/absence of DNA from Gd on each sample of wing skin (within the defined sensitivity limitations of the technique used), extracted nucleic acid was analyzed by PCR as previously described by Lorch et al. [24].

Wing membrane from these same animals was also analyzed by histology [5] to determine WNS infection status. The entire wing membrane was stripped from the right forearm and digits, rolled onto 2 dowels 2.5 cm in length, trimmed into three approximately 0.8 cm-wide sections, placed on trimmed edge, sectioned at 0.4 µm-thickness, and stained with Periodic Acid Schiff [5]. This preparation technique yields six whorls of wing membrane on each slide. White-nose-syndrome was diagnosed based on previously published microscopic criteria [5]. A histologic scoring system was developed to classify severity of WNS on a scale of 0 to 4 as described and illustrated in Appendix S2. Briefly, a score of 0 indicates the sample is negative for WNS, and there are no diagnostic cupping erosions in the tissues. A score of 1 indicates the tissues are positive for WNS with cupping erosions diagnostic for WNS but erosions are mild, occasional, and are limited in both depth and extent of wing membrane involved. The presence of even one characteristic WNS erosion is sufficient for a diagnosis of WNS. A severity score of 2 indicates moderate WNS with more frequent and deeper fungal cupping erosions diagnostic of WNS, but distribution over wing membrane is still limited. A WNS severity score of 3 indicates moderately severe fungal infection with deeper and coalescing cupping erosions that are deep enough to be considered ulcers, and the extent of the wing membrane with fungal invasion is greater. A severity score of 4 indicates a severe fungal infection with deep tissue invasion and coalescing of cupping erosions; as many as 100 or more erosions/ulcers can be present in one roll of wing membrane. Scores ranging from 1 to 4 were identified as WNS.

### Analyses

#### Calculations and initial statistics

Usable data for our analyses were recovered from 99 of the 504 loggers deployed (see Table 1). Although data downloaded from 111 loggers, data from 12 of these bats were removed from final analyses for a variety of reasons, including having temperature data recorded for too short of a time period to be comparable to other groups and missing body mass data. Prior to datalogger attachment, each bat was weighed using a portable battery-operated scale (accuracy to 0.1 g), and the length of their right forearm was measured (in triplicate) to the nearest mm using calipers; from these data BMI (weight in g/length of right forearm in mm) [10] was calculated. As most analyses included BMI as a covariate, only bats for which we were able to calculate BMI at the beginning of hibernation (November) were included in the final analysis (N = 83). Data from an additional 16 bats for which we had recordings from only January through March (see Table 1) are also described in the results.

Torpor was defined as when a bat’s T_sk_ was 10°C or more below its highest temperature (T_max_). Duration of an arousal episode (when T_sk_ was within 10°C of T_max_) was calculated to the nearest 30 min. Although recording T_sk_ every 30 min was sufficient to detect arousal episodes, it did not provide sufficient resolution to describe precisely the true length of an arousal bout, as arousal episodes averaged less than 90 min in length (see results). Thus, we did not attempt to determine if there were significant differences in arousal episode length by WNS status. Torpor bout length (TBL, in days) was defined as the period between two arousal episodes. For both arousal bout length and TBL, values were first averaged for each bat and then averaged across all bats. Data on TBL were log_(10)_ transformed to achieve normality and homogeneity of variance, as determined by Shapiro-Wilk’s test for normality and examination of skew and kurtosis and by Levene’s test for equality of variances. BMI data were normally distributed. TBL data from multiple years are combined in our analysis, which is supported by the lack of a year-to-year difference in TBL in bats from a given hibernaculum when the WNS status did not change between years (e.g., from site 6 (Table 1; Figure 1): 10.52±1.62 days (2008–2009) vs. 12.47±3.09 days (2009–2010); F_(1,16)_ = 3.091, p = 0.098; partial eta squared  = 0.162, power  = 0.380). For all analyses, power and effect size are reported for non-significant results. All data are presented as the mean ± standard deviation (SD).

#### WNS status and TBL

For the initial analysis, bats for which we had data on TBL, BMI, and sex were grouped into three ‘WNS status’ categories: (1) unaffected [N = 57], (2) WNS-affected (as determined by histology and/or visible fungus) and alive at time of datalogger removal [N = 14], and (3) WNS-affected and found dead at time of datalogger removal [N = 12]. Bats were assigned to the ‘unaffected’ category either when the presence of fungal infection with Gd was not detected with PCR or histology [N = 10] or when they were from a hibernaculum presumed to be unaffected and not located in the WNS zone at the time of study [N = 47] (Fig. 1). Combining the two groups of ‘unaffected’ bats for further analyses is supported by the lack of a difference in TBL between them (17.55±4.56 days (PCR/histology) vs. 16.06±7.03 days (presumed unaffected); F_(1,55)_ = 1.111, p = 0.297; partial eta squared  = 0.020, power  = 0.179). Effects of WNS status on TBL were tested with ANCOVA, with BMI (random), site identity (fixed), and sex (fixed) as covariates. Post-hoc examination of sex differences in BMI was conducted using a Student’s *t*-test (with df and p values adjusted for unequal variance).

#### TBL and date of death

Within the WNS-affected bats that were found dead at the time of datalogger removal, the relationships between TBL and BMI and date of death were analyzed using Pearson Product Moment Correlations (PPMC) (after confirming normality and homoscedasticity for each variable). Date of death was measured as the date on which T_sk_ <0°C for the first time, since the T_sk_ of little brown bats always remains above 0°C during torpor [17], [18]. P values were adjusted for multiple comparisons using sequential Bonferroni correction [25], and the coefficient of determination (r^2^) was calculated by squaring significant correlations.

#### TBL and WNS severity score

Using a subset of animals for which a ‘WNS severity score’ could be calculated and for which BMI at the start of hibernation was available (N = 26), the effects of severity score, BMI, and site on TBL were examined with ANCOVA. A significant relationship between severity score and TBL was examined using the Gamma Correlation Statistic, which allows for multiple ‘tied rankings’ [26]. Of these 26 bats, 10 were classified in the first analysis as “unaffected” 13 were classified in the first analysis as “WNS-affected and alive at time of datalogger removal” (of these three bats received a severity score of 1, four bats a severity score of 2, two bats a severity score of 3, and four bats a severity score of 4), and three were classified in the first analysis as “WNS-affected and found dead at time of datalogger removal” (of these two bats received a severity score of 2 and one bat a severity score of 3).

## Results

### Arousing to Euthermic Temperatures

During the course of this study, when bats aroused from torpor, they remained at euthermic temperatures for a short period, averaging 78.3±27.3 min. The range of average arousal bout length per bat was from 38.18 to 180 min (N = 83 bats), while the shortest recorded arousal bout lasted 30 min (the shortest period that could be discerned by our methods) and the longest 330 min. We were unable to test for differences in arousal bout length in relation to WNS status (or severity score) due to the limited data storage capacity of our dataloggers (and thus insufficient resolution for precisely quantifying arousal bout length).

### WNS Status and TBL

Although female bats were in significantly greater body condition than males at the start of hibernation (BMI: 0.2284±0.0283 g/mm (N = 32) vs. 0.2073±0.0210 g/mm (N = 51); t = −3.633, adjusted df  = 52.2, p = 0.001), there were no detectable influences of sex on TBL (F_(1,76)_ = 0.031, p = 0.861; partial eta squared  = 0.000, power  = 0.053). Likewise, we did not detect a relationship between BMI at the start of hibernation and TBL (F_(1,76)_ = 0.140, p = 0.710; partial eta squared  = 0.000, power  = 0.066). Our BMI analyses were not biased by recapture dynamics as there was no significant difference in BMI at the time of datalogger attachment between bats for which loggers were retrieved and bats that were not recovered (Mann-Whitney U = 3.339, Z = 1.259, p = 0.208). However, both WNS-status and site identity significantly influenced TBL. Site identity heavily influenced the model (F_(1,78)_ = 25.027, p<0.001) as two of the sites contained only one category of bat (site 1 had only ‘WNS dead at time of datalogger removal’ bat, and site 6 had only ‘unaffected’ bats). Despite the strong influence of site identity, a significant WNS status main effect was still apparent (F_(1,78)_ = 7.569, p = 0.007).

**Figure 2:**
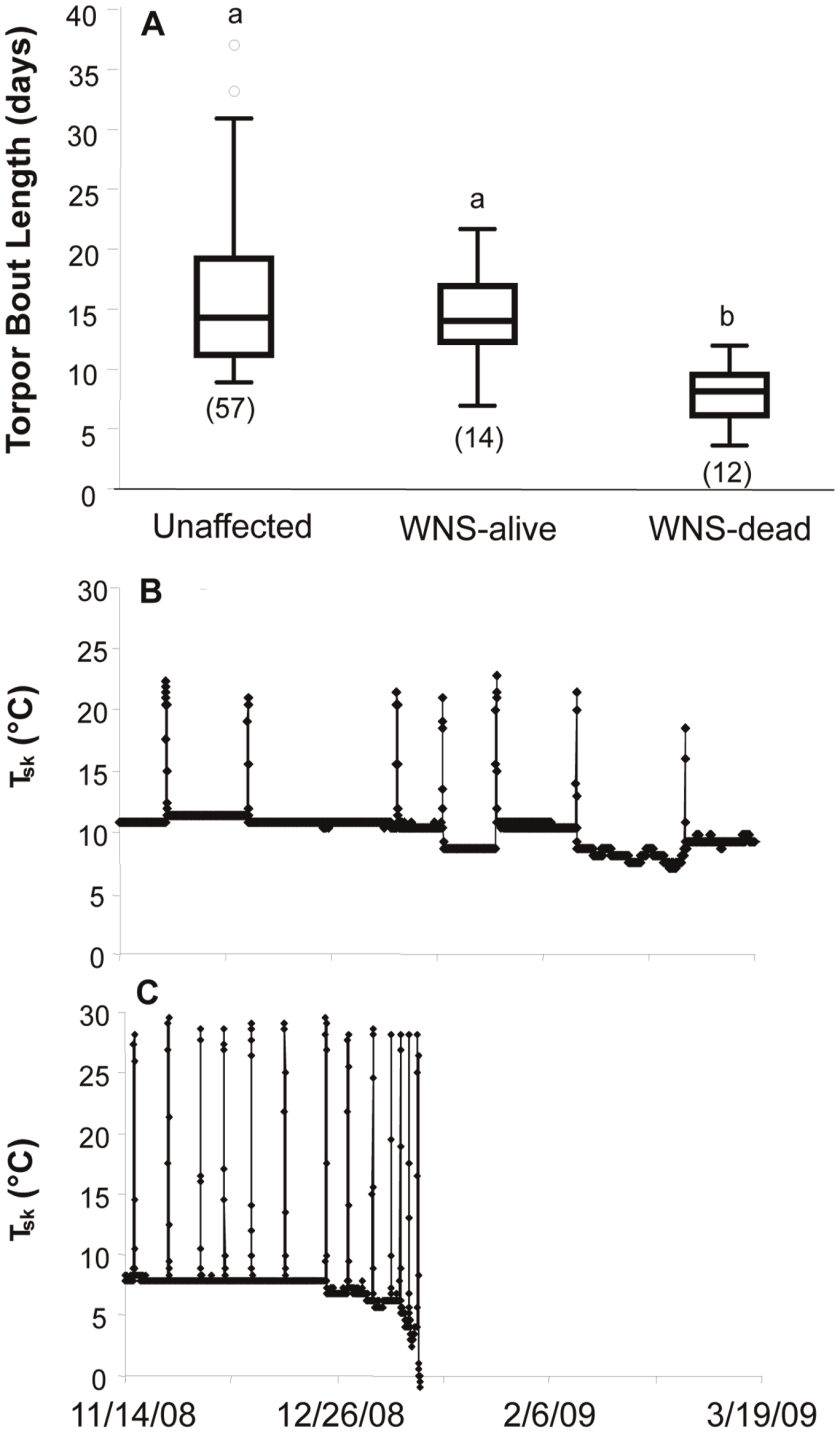
Torpor bout length (TBL) in days by WNS status. WNS was associated with decreased TBL: bats that were affected by WNS but still alive at the collection of dataloggers (March) had shorter TBLs than unaffected bats (but this difference was not significant). Significantly shorter TBLs were seen in WNS-affected bats that were found dead at the time of datalogger collection compared to affected but alive bats (2A). Bats were categorized as: unaffected, WNS-affected and alive at time of datalogger removal (‘WNS-alive’), and WNS-affected and dead when loggers were removed in the spring (‘WNS-dead’). Numbers in brackets indicate sample size and boxes sharing the same letter are not significantly different from each other. Boxes depict the 25th and 75th percentiles, lines within boxes mark the median, and whiskers represent 95th and the 5th percentiles. Outliers are indicated with open circles. Additional panels illustrate sample temperature profile of an unaffected (B) and an affected (C) bat, during the winter of 2009. The bat illustrated in C displayed daily arousals at the end of its life, which was seen in several of these animals. Each of the ‘WNS-dead’ bats died at the end of their last arousal.

Unaffected bats had a mean TBL of 16.32±6.65 days (Fig. 2). Limited data collected from an additional 12 unaffected bats from field sites where dataloggers were deployed for only eight weeks late in the hibernation season in 2009 are similar with a mean TBL of 15.62±8.07 days (sites 2 and 5, Fig. 1). As predicted, having WNS was associated with decreased TBL (Fig. 2). Bats that were affected by WNS but still alive at the collection of dataloggers (March) had shorter TBLs than unaffected bats, although the difference was small and not statistically significant (13.96±4.30 days vs. 16.32±6.65 days; F_(1,69)_ = 1.491, p = 0.226, partial eta squared  = 0.021, power  = 0.226). However, these affected but alive bats had significantly longer TBLs than WNS-affected bats that were found dead at the time of datalogger collection (7.93±2.49 days; F_(1,24)_ = 17.191, p<0.0001). Limited data collected from an additional four WNS-affected bats found dead from a field site where dataloggers were deployed for only eight weeks late in the hibernation season in 2010 are similar with a mean TBL of 6.17±1.79 days (site 4, Fig. 1).

### TBL and Date of Death

**Figure 3:**
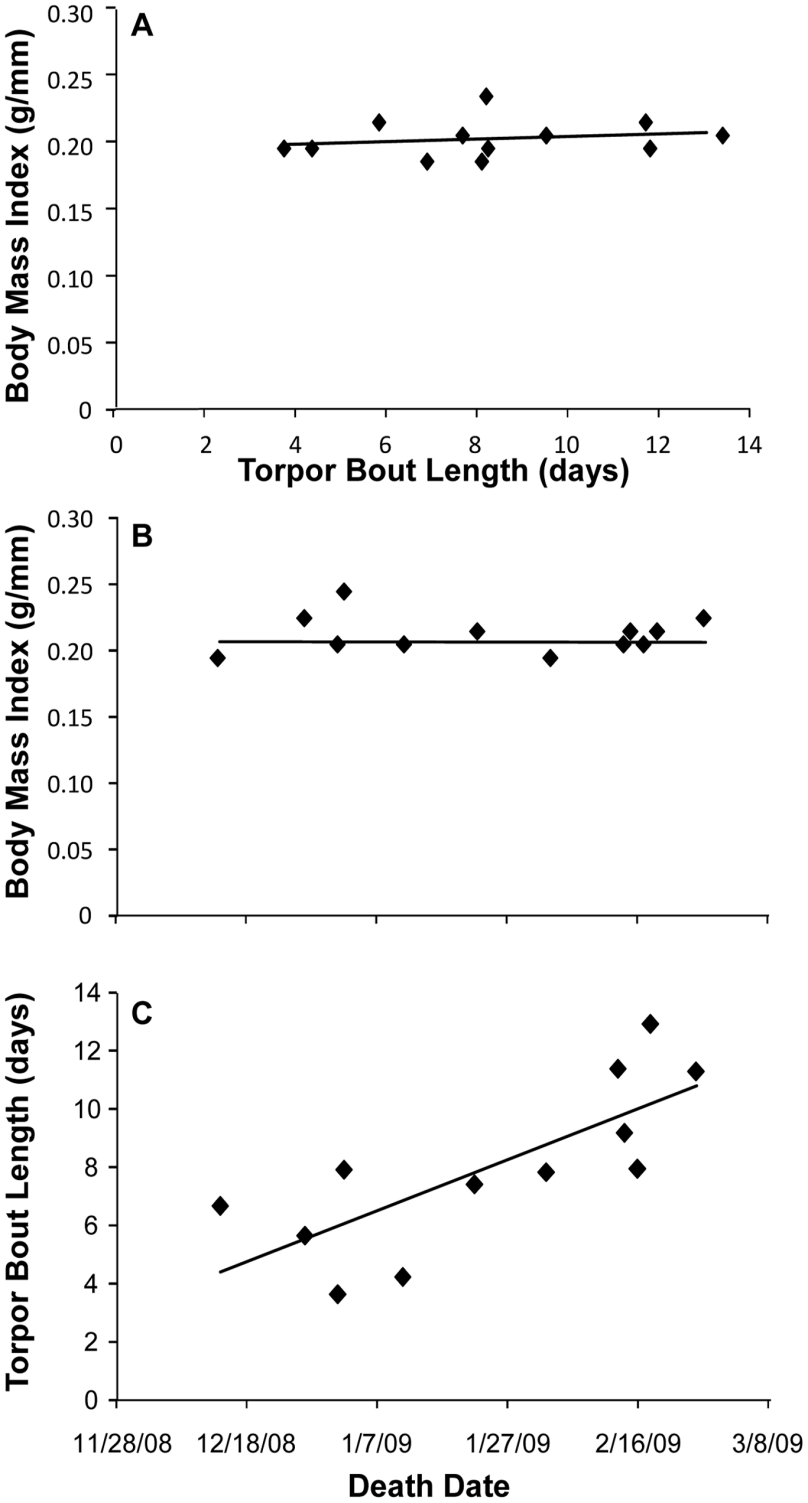
Torpor bout length (TBL) as a function of date of death and BMI. For the 12 bats that died from WNS, BMI at the beginning of hibernation was not related to TBL (3A), nor was BMI predictive of the date of death (3B). However, TBL significantly predicted date of death in WNS-affected bats that were found dead at the time of datalogger retrieval (3C) (r^2^ = 0.58). Bats that died sooner were arousing to euthermic temperatures much more frequently than those that lived longer.

Within the 12 WNS-affected bats found dead at the time of datalogger collection, there was a very strong positive relationship between TBL and the number of days that a bat lived (Fig. 3; PPMC, r = 0.763, corrected p = 0.012). Based upon the calculated coefficient of determination (r^2^ = 0.582), TBL significantly predicted the date of death, explaining 58% of the variance. Similar to the findings of our full ANCOVA, we did not detect a relationship between BMI at the start of hibernation and TBL (PPMC, r = 0.178, p = 0.580) or between BMI at the start of hibernation and date of death (PPMC, r = −0.026, p = 0.936). While the power to detect significant differences at these low effect sizes (correlation coefficients of 0.178 and 0.026) is extremely low (<0.05), even if they were statistically significant, they are not biologically significant. In each bat, mortality was observed immediately after the last arousal to euthermic temperatures. While several bats (Fig. 2C) displayed frequent arousals just before death, most did not, and arousals were spread throughout their hibernation period.

### TBL and WNS Severity Score

**Figure 4:**
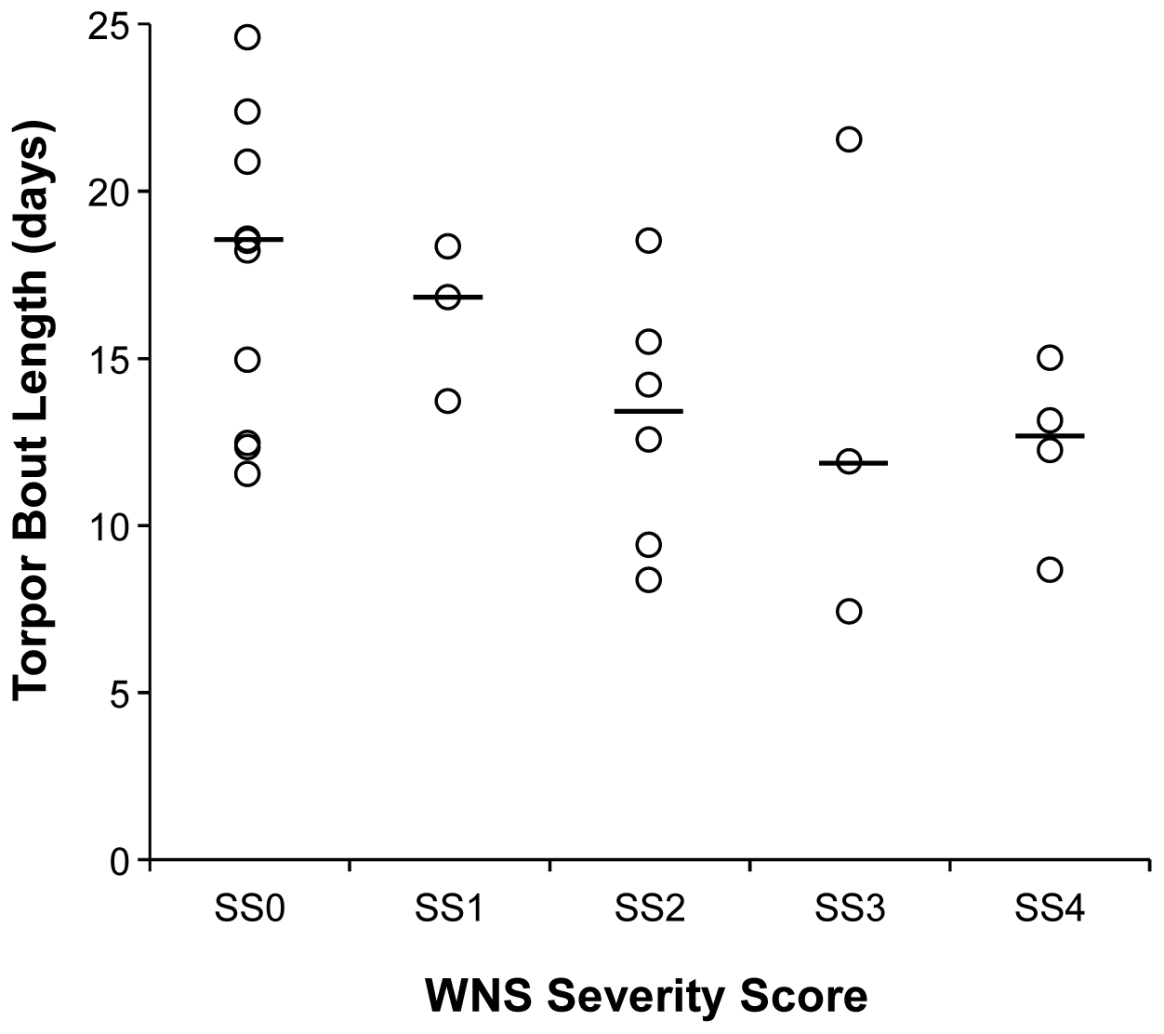
Torpor bout length (TBL) as a function of WNS severity score. Wing tissue was assigned a disease severity score (SS0 to SS4) based upon histology, as follows: SS0 =  no fungi suggestive of WNS; SS1 =  occasional but limited superficial fungal infection; SS2 =  more extensive superficial fungal infection with limited invasion; SS3 =  more extensive fungal infection with frequent cupping erosions; and SS4 =  severe fungal infection with deep tissue invasion. Details of the scoring system can be found in Appendix S2 and scores 1 through 4 were identified as WNS. Individual data points are shown as open circles, the median is indicated by a line. As severity of infection increased, torpor bout length significantly decreased (bats aroused more frequently from torpor.

In the subset of animals for which the WNS severity score could be calculated (N = 26), TBL was not related to BMI (F_(1,21)_ = 0.111, p = 0.743, partial eta squared  = 0.005, power  = 0.062) or site identity (F_(2,22)_ = 2.515, p = 0.104, partial eta squared  = 0.186, power  = 0.045), but was related to severity score (F_(1,24)_ = 6.509, p = 0.018). Bats with more severe fungal infections had significantly shorter torpor bouts (gamma correlation statistic  = −0.383, p = 0.022; Fig. 4).

## Discussion

Our results support the hypothesis that WNS causes alterations in bat torpor patterns that likely contribute to death. Our prediction that increased mortality/disease state is associated with abnormally short torpor bouts due to frequent arousal episodes was supported by our larger dataset, in which bats were placed into the WNS status categories of ‘unaffected,’ ‘WNS-affected and alive at time of datalogger collection at the end of hibernation,’ and ‘WNS-affected and dead at the time of datalogger collection.’ While our ‘unaffected’ bats had an average TBL that falls within the previously documented range for this species (16.32 days) [16], [17], TBL was shortened (at the low end of previously described TBLs) in WNS-affected bats (13.96 days), and significantly reduced in WNS-affected bats that died between mid-December and late-February (7.93 days). An average torpor bout length of 7.93 days is presumably not sustainable. In fact, within those WNS-affected bats found dead at the time of datalogger removal, TBL was a very strong predictor of the date of death, explaining 58% of the variance in timing of mortality. The distribution of death dates for these bats (Fig. 3) is earlier than that reported in the USA [7] and earlier than seasonal changes in Gd prevalence reported for Europe [27], [28]. However, this was at least the second year of infection at this site, which might shift the distribution of death dates earlier relative to compiled data from multiple sites [7], [27], [28]. Recapture of bats for datalogger removal in March of each year (Table 1), the time when peak mortality has been noted in the field [7], may have prevented us from detecting other mortality events within our study animals.

Our analysis of WNS severity based upon histological confirmation of the degree of fungal invasion and infection further supported and strengthened our conclusion – as the severity of infection increased, so did the frequency of arousals from torpor. Our data mirror the independently derived mathematical model of Boyles and Willis [9], for which an estimated shift in TBL to every 8.33 days resulted in a prediction of 81.9% mortality. Relative to this model, our finding of a TBL of 7.93 days for WNS-affected bats found dead, and field observations of 91% mortality support the linkage between TBL and death, as significant body fat is lost with each arousal [13], [18]. Boyles and Willis [9] also proposed that significant changes in arousal bout duration in WNS-affected bats could lead to mortality. Bats are unlike other hibernators [13], [18] in that their arousal bouts are typically measured in minutes rather than hours (or even days). Thus, an increase in the duration of euthermy would incur significant energetic costs. Although we were unable to statistically validate differences in arousal bout length in bats of variable WNS status, our finding of an average arousal bout of 78.3±27.3 minutes for all bats tested indicate that biologically important shifts in arousal bout length do not occur in WNS-affected animals.

We also predicted that relationships between WNS and torpor patterns would be influenced by the amount of energy stores available to the bat. In a previous study of little brown bats, BMI significantly influenced hibernation energetics such that bats with lower body masses at the beginning of hibernation selected colder roosting sites, which allows for decreased metabolic rates and thus lower energy expenditure [29]. Other studies have demonstrated that bats roosting at colder temperatures arouse from torpor less often, allowing them to conserve even more energy [19], [30], [31]. Thus, it is reasonable to expect that bats with lower BMIs would display greater TBL and expend less energy.

These energetic arguments underlay the model of Boyles and Willis [9] that our data so closely match. However, contrary to our predictions, we did not find a relationship between BMI and TBL or BMI and ‘WNS status’, death date, or ‘severity score’. As the power for BMI effects in our models was low (driven by the strong site effects), BMI may still play a role in hibernation patterns and in a bat’s ability to withstand Gd infection. However, even within a site (WNS-affected bats that were found dead at the time of datalogger attachment from site 1 in Vermont), we failed to find a relationship between BMI and WNS. If a higher BMI could ‘buffer’ a bat from the effects of WNS by allowing it to withstand more arousals to euthermy, then we should have detected a relationship between BMI and the number of arousals prior to death – but we did not.

Although statistical analyses confirmed the significance of our findings, studies of behavior and physiology in free-ranging animals are often fraught with unknowns and potential biases, which likely underlie the significant site effects in our statistical models. One potential source of bias in our dataset is BMI at the start of the hibernation season. While one could predict that bats in poorer body condition would find datalogger attachment more physiologically stressful than bats in greater body condition (and thus be less likely to be recaptured), there was no difference in starting BMI between bats that were recaptured and those that were not. Another source of bias in our WNS-affected bats could have been ambient temperature of hibernacula, because TBL generally decreases with increased ambient temperature [30]. Although the exact ambient temperature at the exact roosting site of each individual studied during hibernation was unknown, our WNS-affected field sites were generally colder than our unaffected sites (e.g., 7.29°C vs. 9.77°C). This would presumably bias bats with WNS toward longer TBLs, but we observed the opposite pattern. Within our unaffected bats, TBLs varied greatly (Fig. 2A), likely due to a number of site-, individual-, and population-specific factors. However, these factors appear to be overridden in the WNS affected bats, especially those found dead at the time of datalogger removal – as variability decreased and all bats exhibited shortened TBLs.

Collectively, our data indicate that one proximate mechanism of the mortality associated with WNS is decreased TBL. Warnecke et al. [21], in a study of captive bats experimentally infected with Gd during the third year of our field study, found a similar TBL shift. The challenge that lies before us is to determine how infection by Gd induces altered torpor patterns and why significant variation in TBL between affected bats occurs. While too-frequent arousal is clearly associated with WNS, not all bats that died displayed the severely shortened TBL characteristic of some that died, and some bats that displayed very short TBL did not die.

In other mammalian hibernators, mechanisms associated with immunity are reduced during hibernation, when the conservation of energy is critical [32], [33], and the periodic arousals from hibernation may activate the dormant immune system. Thus, immunological responses to fungal infection may be triggering arousals more frequently than normal [34]. Additionally, physical damage to wing skin caused by fungal infection may disrupt other physiological functions, such water balance, resulting in dehydration, another trigger for arousal from torpor in hibernating animals [8]. Equally important to understanding how Gd infection leads to altered torpor patterns is the need to understand how these too-frequent arousals to euthermy may be contributing to death – in ways that are not clearly related to energy balance, but are potentially related to the disruption of other homeostatic mechanisms [8].

A detailed understanding of the mechanism(s) by which infection with Gd causes mortality in hibernating bats may provide insights to develop interventional strategies to mitigate this unprecedented wildlife disease. Insectivorous bats perform significant ecosystem services because they are primary predators of nocturnal insects [35]–[37]. As such, we believe that the loss of cave-dwelling hibernating bats in North America will be ecologically significant.

## Supporting Information

Appendix S1 Instructions for producing temperature sensitive dataloggers for attachment to bats, including figures.
(PDF)

Appendix S2 Description of WNS histopathology and assignment of wing damage severity scores (SS), including figures.
(PDF)
