# Supplementary material for: Frequent Arousal from Hibernation Linked to Severity of Infection and Mortality in Bats with White-Nose Syndrome
Source: PLoS One. 2012 Jun 20;7(6):e38920. doi: 10.1371/journal.pone.0038920 (PMC3380050; doi:10.1371/journal.pone.0038920)
Supplement: Appendix S1 — Instructions for producing temperature sensitive dataloggers for attachment to bats, including figures. (PDF) [file pone.0038920.s001.pdf]

Frequent Arousal from Hibernation Linked to Severity of Infection and Mortality in Bats with White-nose Syndrome.

DeeAnn M. Reeder[a*], Craig L. Frank[b], Gregory G. Turner[c], Carol U. Meteyer[d], Allen Kurta[e], Eric R. Britzke[f], Megan E. Vodzak[a], Scott R. Darling[g], Craig W. Stihler[h], Alan C. Hicks[i], Roymon Jacob[a], Laura E. Grieneisen[a], Sarah A. Brownlee[a], Laura K. Muller[d], and David S. Blehert[d]

[a]Department of Biology, Bucknell University, Lewisburg, PA, USA; [b]Department of Biological Sciences, Fordham University, Armonk, NY, USA; [c]Pennsylvania Game Commission, Harrisburg, PA, USA; [d]U.S. Geological Survey - National Wildlife Health Center, Madison, WI, USA; [e]Department of Biology, Eastern Michigan University, Ypsilanti, MI, USA; [f]US Army Engineer Research and Development Center, Vicksburg, MS, USA; [g]Vermont Fish and Wildlife Department, Rutland, VT, USA; [h]West Virginia Division of Natural Resources, Elkins, WV, USA; [i]New York State Department of Environmental Conservation, Albany, NY, USA

Supporting Information: Appendix S1. Instructions for producing temperature sensitive dataloggers for attachment to bats.

Temperature sensitive external dataloggers (Bucknell University Temperature Trackers, BUTTs) were made in from Thermochron DS1922L iButtons (Maxim Integrated Products, Inc., California, USA), which is the same underlying technology found in AlphaMach's iBBat dataloggers (AlphaMach, Inc., British Columbia, Canada). The protocol for creating, programming, and calibrating the iButton components is based upon the modification and miniaturization procedure as described by Lovegrove [S1], but has marked differences.

The DS1922L iButtons are self-contained cylindrical temperature loggers that can measure and record temperatures from -40°C to 85°C. A total of 8,192 8-bit readings can be recorded at periodic intervals ranging from 1 second to 273 hours. Each logger has a unique serial number, which is located on the can and is displayed in the program when missioning the logger. The user-defined mission can start immediately, start upon a specific time delay, or start once a specified temperature has been reached.

OBTAINING THE INTERNAL COMPONENTS FOR BUTT CONSTRUCTION:

De-house the iButton: Each iButton weighs 3.3 grams and is made with a silicon chip and battery contained in a stainless steel casing or "can," consisting of a "lid" (part with writing) and "base" (cylinder sides and lipped face) (Fig. S1A). BUTTs only utilize the chip and battery, and thus it is necessary to extract these components. This is accomplished by sawing the casing with a flat metal hand-file approximately one-third to halfway (~ 2:30 and 9:30 on a clock face) on each side of an iButton clamped in a vice (Fig. S1B). File the case at the two points until a black plastic grommet is exposed at the lid edge and there are clear cuts on the lip of the base. While still in the vice, use a set of pliers or the top of the file to bend back the base from the lid (Fig. S1C). After removing the iButton

from the vice, the lid of the canister can be removed and the inside components can be extracted (Fig. S1D).

Clip Grommet, Circuit Board, and Input/output Prong: The inside components consist of the silicon chip and battery surrounded by a black plastic grommet. On the chip a three-pin terminal is found consisting of battery negative, battery positive, and input/output (I/O) terminals. To reduce the weight of the final datalogger, some of these components can be trimmed. Separate the circuit board from the battery by sliding it away from the pins. Use clippers to cut and remove the black plastic grommet to the left and right of the terminal (Fig. S1E-F). Small sharp scissors may be used to trim the chip about 2 mm and 0.5mm from the left and right, respectively, as pictured (Fig. S1G), taking special care not to clip off important components.

To further reduce weight and to prevent future failures when extracting data from the loggers, it is possible to cut the longest prong (I/O) (as shown in Fig. S1H). Use a pair of small sharp scissors and cut it to a length similar to the other prongs. The length of this prong often makes the circuit board detach from the battery when extracting the internal components from their plastic coating for data downloading (see below).

Glue Circuit Board to Battery and Epoxy the Pins: If at any point a programmed circuit board loses its connection with the battery via the three prongs, the mission and all of its recorded data will be lost. To reduce the likelihood of data loss, glue the chip to the battery with a dab of fast setting five minute epoxy (e.g., Locite Instant Mix Epoxy, Loctite® Brand Consumer Products, Henkel Corporation, Westlake, Ohio, USA), and connect the pins to the circuit board with a special flexible silver-filled conductive epoxy (McMaster-Carr, Cleveland, OH, USA; item # 7661A13). This glue comes in small easy-mix packets (2.5 grams).

To glue the circuit board to the battery, mix a small amount of equal parts epoxy resin and five minute hardener (dispensed from a dual syringe) with a wooden applicator stick. Use the stick to spread a thin layer in the center of the battery, making sure not to spread too close to the black grommet that holds the terminal pins. Slide the chip onto the battery to align with the prongs, and then lightly press the circuit board to battery to ensure a bond will form (Fig. S1H). Allow the glue to dry in a fume hood or other ventilated area.

For connecting the pins to the circuit board, mix a small amount of equal parts of the two components of the silver-filled epoxy with a syringe needle and apply a tiny, but sufficient, dot on the far left and right connection points, avoiding the middle prong (shown in Fig. S1H). This is best done under a dissection scope, in order to ensure that the applied silver glue does not touch between any two connection points (which would stop the logger from functioning). Lovegrove [S1] recommends soldering the pins to the circuit board, but we find the use of electrically conductive glue sufficient, and easier.

FINAL BUTT PREPARATIONS:

Program: The modified chip and battery now can be prepared for programming and coating. BUTTs are constructed from the pieces of the iButton and are thus programed using the same software (One Wire Viewer: http://www.maxim-ic.com/products/ibutton/software/1wire/OneWireViewer.cfm). The iButton reader does not work without the canister, however, so a specially modified lead system must be made. As Lovegrove [S1] explained, the connecting lead should be created from a 6P2C modular (telephone) cord or Ethernet cord that is appropriate for the 1-Wire RJ11 port reader. Instead of using crocodile clips, we have found that splicing probe leads to the cable eliminates the risk of pulling the leads off of the circuit board. For initial programming, one person touches the probe leads to the I/O and ground pins on the circuit board while a second person types missioning specifications into the computer. Alternatively, for the initial programming (but not later downloading), the probe leads can be propped in place with larger clips and balanced on the table, allowing a single person to program the loggers (Fig. S1I). After the logger is programmed and before it is covered in plastic coating, it is important to not allow de-housed iButtons to touch each other as this can cause a loss of the programming.

String: In order to facilitate coating in plastic, pass about 250 cm total length of thread through the gap between the circuit board and battery on the terminal end of the modified iButton (Fig. S1J). Place a piece of lab tape folded onto itself on one end of the string to record either the serial code of the circuit board or another unique number assigned to the datalogger. The string can be put in place before or after programming. The serial code of the circuit board can be found on the original can that housed it, or is displayed in the missioning software.

Plastic Cling Wrap: The internal components must be wrapped in plastic wrap before they can be coated with a thick plastic dip. The piece of wrap used must cover the components completely, but not add excess surface for the dip to cling. Using a small square piece (about 3.8 X 3.8 cm) of plastic cling wrap, place the logger components face down with the strings up and one corner of wrap between the two strings. Fold the left and right corners to cover the battery first, followed by the bottom corner. Finally, fold the top corner down. Make sure that the strings are pulled taught and the plastic wrap does not bunch on them. By folding the top down last, the chances of the dip pulling the plastic off are minimized (Fig. S1K).

Plasti-Dip: The logger components are now ready to be coated in a synthetic rubber, Plasti Dip, which is available in a variety of colors (Plasti Dip International, Blaine, MN, USA). The number of coats of Plasti Dip is based on lab or field use (thicker for enduring field conditions) and the starting thickness of the liquid Plasti Dip, which thickens in the jar after opening. Pour about 40 mL of Plasti Dip into a

plastic 50 mL beaker. Mix small amounts of turpentine into the Plasti Dip until the appropriate consistency, approximating that of warm honey, is reached. Multiple thin coats are preferable to a single thick coat. Holding onto the strings, dip the logger in its plastic wrap into the Plasti Dip and then hold it over the Dip container to allow excess to rubber to drop off. Hang the logger to dry in a fume hood or other highly ventilated area by both strings (Fig. S1.1L). Before applying additional coats of Plasti Dip, excess plastic can be trimmed (e.g., at the bottom corners).

Label: Once BUTTs are fully dipped and dry, carefully remove them from the fume hood. Clip the strings from the BUTTs, but make sure to keep the ID tag with its logger. Use an indelible marker to write the ID number on the flat, battery side of the BUTT (Fig. S1M), as the side of the logger with the leads is attached to the bat. The number also can be written on the edge of the logger, where the strings were. Unlike the unprotected de-housed iButton components that cannot touch, BUTTs after coating can now touch anything.

Calibrate: Because of the significant alterations that have been made to the iButton components, and because of variable levels of plastic coating on the final BUTTs, each logger must be individually calibrated to ensure accurate measurements. To calibrate loggers, BUTTs are placed in an airtight container with a thermocouple set to record temperature every 15 s, and the container is submerged into 2°C, 23°C, and 37°C water baths for a minimum of 4 hours each. After downloading the values from the thermocouple, an average of at least 20 temperature recordings should be taken from the timepoints in which temperature was stable at each of the calibration temperatures. These averaged values are T_1, T_2, and T_3, respectively. The average temperature recorded when the thermocouple was at each calibration temperature (T_1, T_2, and T_3) can then be compared to the values from identical timepoints for each individual logger to generate three deviations (temperature of logger - temperature of thermocouple; D_1, D_2, and D_3). These deviations are used to find the equation of the quadratic curve, from which additional temperature readings (from the loggers) can be corrected. If the three deviations are D_1, D_2, and D_3 and the temperatures measured by the thermocouple are T_1, T_2, and T_3, the roots of the quadratic equation (α (alpha) and ß (beta)) can be calculated, as can be the constants A, B, and C, as follows:

$$\alpha = (D_1 - D_3) + ((D_2 - D_1)*(T_3 - T_1))/(T_2 - T_1)$$

$$ß = T_3^2 - T_2*T_3 + T_2*T_1 - T_3*T_1$$

$$A = D_1 - B*T_1 - C*T_1^2$$

$$B = (D_2 - D_1 - C*(T_2^2 - T_1^2))/(T_2 - T_1)$$

$$C = - \alpha / ß$$

From these values, corrected temperatures are calculated as follows, where T_M is the uncorrected (measured) temperature and T_C is the corrected (calculated) temperature:

$$T_C = T_M - A - B*T_M - C*T_M^2$$

While data from the thermocouple should be downloaded immediately after calibration to ensure that the thermocouple recorded periods of stable temperature at each of the three calibration temperatures, the remainder of the calibrations must be performed after data from the loggers are downloaded (after removal from a bat).

Download: To re-access the terminal pins needed to download the information from the loggers, carefully make an incision at the top edge of the logger (where the remaining portion of the grommet is). Peel back the PlastiDip and plastic wrap just enough to expose the pins. Establish a connection with the computer and software using the leads as described above in the programming section.

References

S1. Lovegrove BG (2009) Modification and miniaturization of Thermochron iButtons for surgical implantation into small animals. Journal of Comparative Physiology B 179: 451-458.

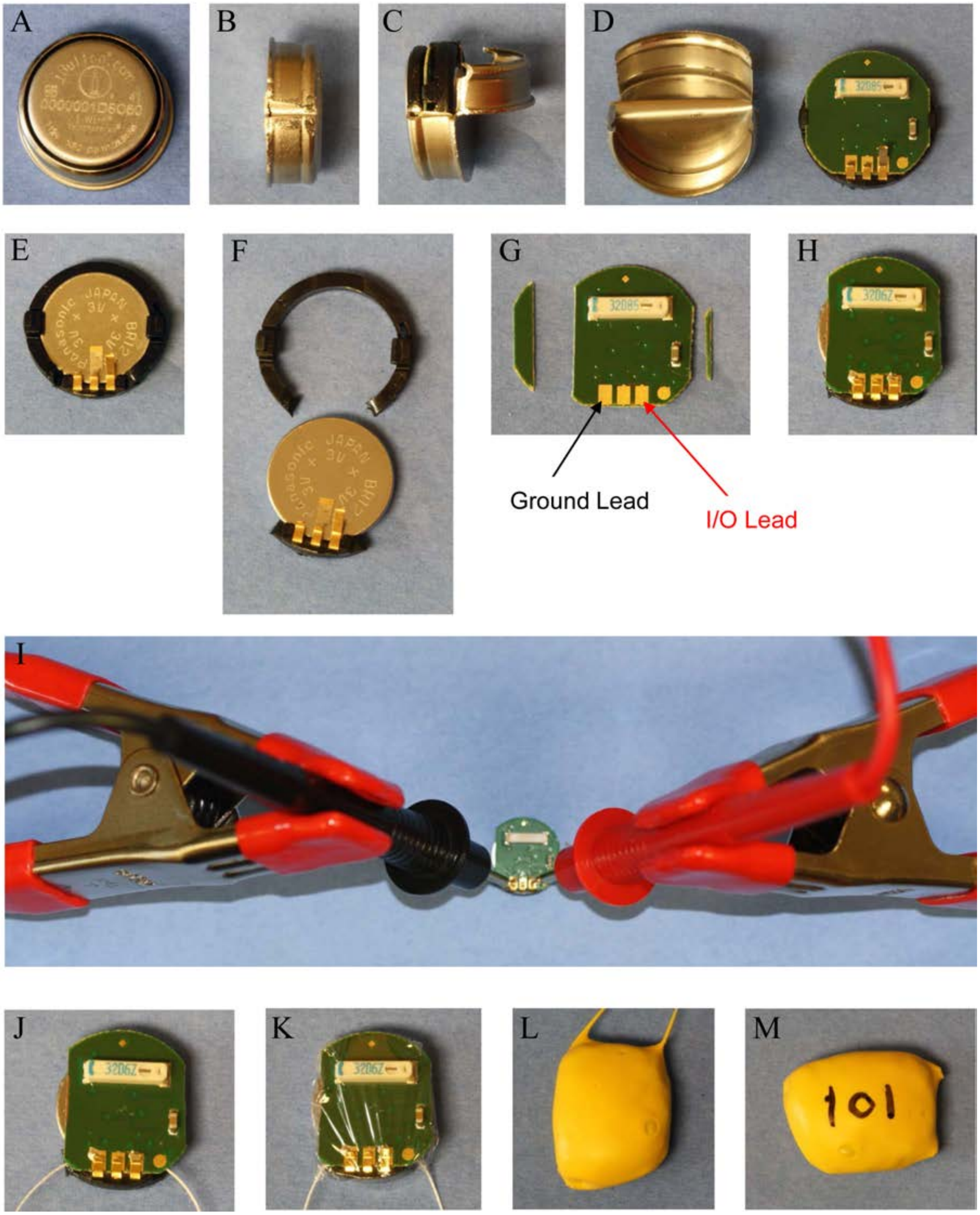

Appendix S1, Figure S1. Construction of Bucknell University Temperature Tracker dataloggers (BUTTs) from iButtons. An iButton prior to modification (A), iButton showing filing to remove casing or 'can' (B), peeling back of can (C), iButton can and internal components separated (D), battery with black plastic grommet intact (E), battery with most of grommet removed to decrease weight (F), circuit board with sides trimmed to decrease weight (G), circuit board reattached to battery, with I/O (input/output) lead trimmed to decrease weight and with leads secured to the circuit board with silver-filled conductive epoxy (H), programming the datalogger via the ground and I/O leads (I), attaching string to facilitate coating in plastic (J), wrapping logger in plastic wrap to protect circuit board from coating rubber (K), first coating in rubber 'Plasti Dip' (L), completed logger (M).
