# Supplementary material for: Frequent Arousal from Hibernation Linked to Severity of Infection and Mortality in Bats with White-Nose Syndrome
Source: PLoS One. 2012 Jun 20;7(6):e38920. doi: 10.1371/journal.pone.0038920 (PMC3380050; doi:10.1371/journal.pone.0038920)
Supplement: Appendix S2 — Description of WNS histopathology and assignment of wing damage severity scores (SS), including figures. (PDF) [file pone.0038920.s002.pdf]

Frequent Arousal from Hibernation Linked to Severity of Infection and Mortality in Bats with White-nose Syndrome.

DeeAnn M. Reeder[a*], Craig L. Frank[b], Gregory G. Turner[c], Carol U. Meteyer[d], Allen Kurta[e], Eric R. Britzke[f], Megan E. Vodzak[a], Scott R. Darling[g], Craig W. Stihler[h], Alan C. Hicks[i], Roymon Jacob[a], Laura E. Grieneisen[a], Sarah A. Brownlee[a], Laura K. Muller[d], and David S. Blehert[d]

[a]*Department of Biology, Bucknell University, Lewisburg, PA, USA;* [b]*Department of Biological Sciences, Fordham University, Armonk, NY, USA;* [c]*Pennsylvania Game Commission, Harrisburg, PA, USA;* [d]*U.S. Geological Survey - National Wildlife Health Center, Madison, WI, USA;* [e]*Department of Biology, Eastern Michigan University, Ypsilanti, MI, USA;* [f]*US Army Engineer Research and Development Center, Vicksburg, MS, USA;* [g]*Vermont Fish and Wildlife Department, Rutland, VT, USA;* [h]*West Virginia Division of Natural Resources, Elkins, WV, USA;* [i]*New York State Department of Environmental Conservation, Albany, NY, USA*

Supporting Information: Appendix S2. Histologic severity scoring (SS) of white-nose syndrome (WNS) using wing membrane.

Wing membrane was used to score the damage associated with WNS. The skin of the muzzle may or may not be affected in bats with WNS, and if infected, may not be as physiologically important as the damage the fungal agent *Geomyces destructans* (Gd) causes to the wing membrane.

All bats used for this classification system were *Myotis lucifugus* that were part of this study or euthanized for state surveillance for WNS, shipped chilled for overnight arrival, and processed the day they arrived to avoid postmortem changes that might interfere with lesion interpretation. It may be difficult to wrap all of the wing membrane on two dowels for bats that are much larger than M. lucifugus. If this is not possible, as much of the leading edge and trailing edge of the wing should be included for histologic evaluation as these margins can be the primary areas infected.

ASSUMPTIONS:

Both wings are equally affected by Gd. Using only one wing for histopathology allows the second wing to be removed aseptically from the body for PCR and culture.

Rolling all of the membrane from one wing onto 2 dowels and trimming each into 3, 0.5 cm segments, embedding and sectioning all segments, mounting on a slides and observing the microscopic sections provides a reasonable representation of severity of wing damage.

Biological systems rarely fit exactly into the round holes we carve out for them, but they can be placed in general categories that can help us better understand disease progression.

METHOD FOR PREPARING WING MEMBRANE:

Dowels are rolled to 0.25 cm diameter from unflavored and uncolored dental orthodontic wax, and cut to 2.5 cm lengths. A piece of orthodontic paraffin rolled to 10 cm long and cut into 4 equal lengths will provide the appropriate length and diameter for the dowels. All membrane from one wing is removed, cut into 1cm strips, rolled in overlapping spirals around the dowel so that all

membrane is wrapped onto 2 dowels resulting in multiple layers of membrane. These paraffin dowels are placed into a labelled cassette to maintain the arrangement of the membrane on the paraffin, and this cassette is placed in formalin for at least 24 hrs. The entire 'membrane roll', inclusive of paraffin dowel, is trimmed to approximately 0.5 cm cross sections yielding approximately 6 whorls of tissue (2 dowels, 3/dowel). These cross-sections of rolls of wing membrane with the central paraffin dowel are placed cut side down in a cassette, processed and embedded in paraffin, sectioned at 4 um, placed on a glass slide and stained using PAS [S1]. Six rolls of wing membrane will be visible on the slide.

METHOD FOR FIGURES S1-S4:

One prototype bat was chosen from this study for each grade; mild, moderate, moderately severe, and severe. The digital images were taken using an Insight Firewire Spot camera and software. One field of view was used for the set of pictures that represent a grade of severity 1 through 4. A set of 4 images was taken at different magnifications to illustrate both distribution (low magnification) and invasion (higher magnifications).

CLASSIFICATION OF SEVERITY SCORES:

Cupping erosions filled with dense aggregates of fungal hyphae are currently used as the criteria to diagnose WNS. Severity scores from 0 (unaffected), to 4 (severe) depend on presence, extent, and distribution of these cupping erosions. The cupping erosions form a discrete interface with the skin. As these erosions progress, the thin, pigmented epidermis is no longer visible at the 'front' of the invading aggregate of fungal hyphae.

Grading the severity of WNS histopathology considers the presence of typical cupping erosions, the depth and surface area of these erosions and the extent to which these erosions cover the observable wing membrane on the slide. If some of the rolls are more severely affected than others on the same slide, the most severely affected wing rolls are used to establish the severity score. It is difficult to assess severity until you have seen a bat wing membrane that truly fits the designation of 'severe'. It is then easier to put the other degrees of severity in perspective.

The degree of fungal surface colonization and production of conidia are not included in the criteria for diagnosing WNS or in the severity scoring system. Colonization of superficial skin with fungal hyphae and production of conidia are quite variable within and between severity grades although, in general, the density and extent of hyphae on the surface and the production of conidia increase with severity.

The presence and degree of inflammation and bacterial infection of wing membranes are not included in the criteria for diagnosing WNS or in the severity scoring system. Bacteria and inflammation are inconsistent findings and are not necessary for full manifestation of WNS and mortality. However, both can be present in some bats, particularly in spring near the end of hibernation.

CRITERIA USED TO ASSIGN SEVERITY SCORES

Severity Score 0 (SS0)
No fungal cupping and erosion; the wing membrane is considered negative for WNS.

Severity Score 1 (SS1) - Mild wing membrane damage with cupping and erosions diagnostic of WNS are present but few (Fig. S1).
Degree of fungal erosion: The cupping erosions are discrete but relatively shallow.
Extent of fungal erosion: Erosions are few and widely scattered over the rolled sections of wing membrane. Even if infection is limited to only one visible 'cupping erosion' in the 6 whorls of wing membrane, it is considered positive for WNS.

Severity Score 2 (SS2) - Moderate wing membrane damage (Fig. S2).
Degree of fungal erosion: Cupping erosions are still separate and relatively discrete, but individual erosions involve tissues deeper in the dermis, can be considered ulcers, and can begin to replace regional adnexa.
Extent of fungal erosion: Usually all rolls of wing have at least some cupping erosions. A minimum of 4 of the 6 wing rolls should have the characteristic erosions. The majority of individual wing rolls usually have approximately 10 or more cupping erosions

Severity Score 3 (SS3) - Moderately severe wing membrane damage (Fig. S3).
Degree of erosion: The dense aggregates of fungal hyphae invade wing membrane replacing the components of dermis, including adnexa. This invasion can become almost trans-membrane and individual erosions and ulcers begin to coalesce, resulting in larger regions of wing membrane that are eroded and ulcerated. Individual hyphae penetrate the deeper dermis beyond the discrete interface of the dense aggregate.
Extent of erosion: All rolls of wing (6/6) have characteristic erosions/ulcers. The majority of individual wing rolls have more than 10 cupping erosions/ulcers and at least 2 rolls should have more than 20.

Severity Score 4 (SS4) - Severe wing membrane damage (Fig. S4).
Degree of erosion: There is extensive tissue invasion. The fungal aggregates coalesce and erode deeper, some almost trans-membrane, and individual hyphae penetrate randomly into the dermis beyond the interface of the fungal aggregate. The morphology of the wing membrane becomes multifocally distorted in response to the extensive fungal invasion. Adnexa can be completely effaced by fungal hyphae and regions of membrane can have changes suggesting infarcts with hypereosinophilia and loss of all identifiable vital structures in the dermis [S2].
Extent of erosion: All of the wing rolls (6/6) have cupping erosions. Most of the rolls have more than 20 erosions and some can have as many as 100 or more.

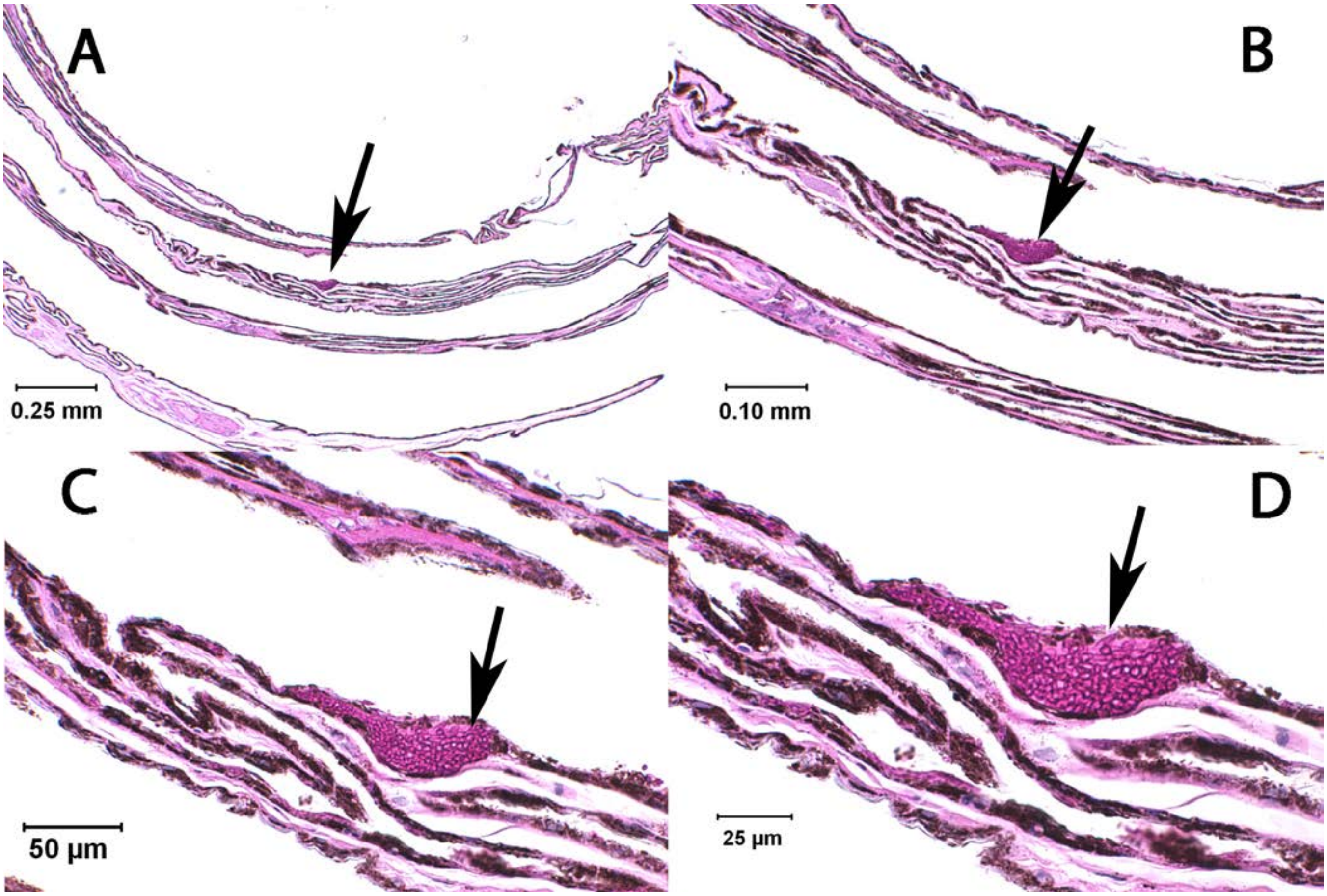

Appendix S2, Figure S1. Wing membrane damage severity score = 1 (SS1), mild damage due to WNS. Photomicrographs of periodic acid Schiff-stained 4-μm sections of wing membrane prepared as described above. A portion of a single roll of wing membrane from a little brown bat (*Myotis lucifugus*) contains a single cupping erosion (arrows) fulfilling the diagnostic criteria for WNS. Four magnifications of this single aggregate (A, B, C, D) have calibrations bars embedded in the image.

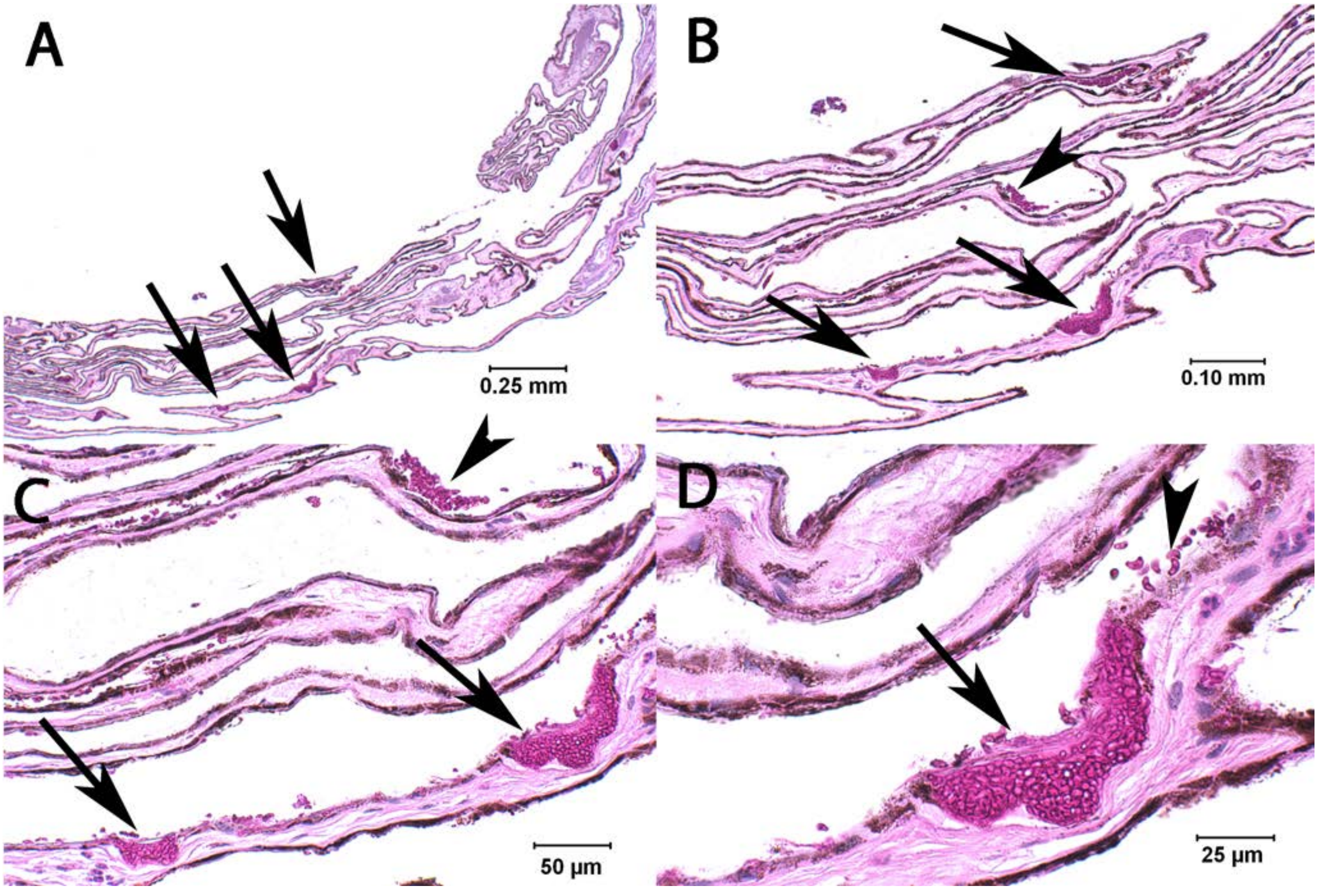

Appendix S2, Figure S2. Wing membrane damage severity score = 2 (SS2), moderate damage due to WNS. Photomicrographs of periodic acid Schiff-stained 4-μm sections of wing membrane prepared as described above. A portion of a single roll of wing membrane from a little brown bat (*Myotis lucifugus*) contains many cupping erosions (arrows). Although more numerous, the cupping erosions are still separate and relatively discrete. Individual erosions are larger than in Fig. S1 and begin to distort the morphology of the wing membrane. Conidia consistent with *Geomyces destructans* are present (arrowheads). Four magnifications of this field of view (A, B, C, D) have calibrations bars embedded in the image.

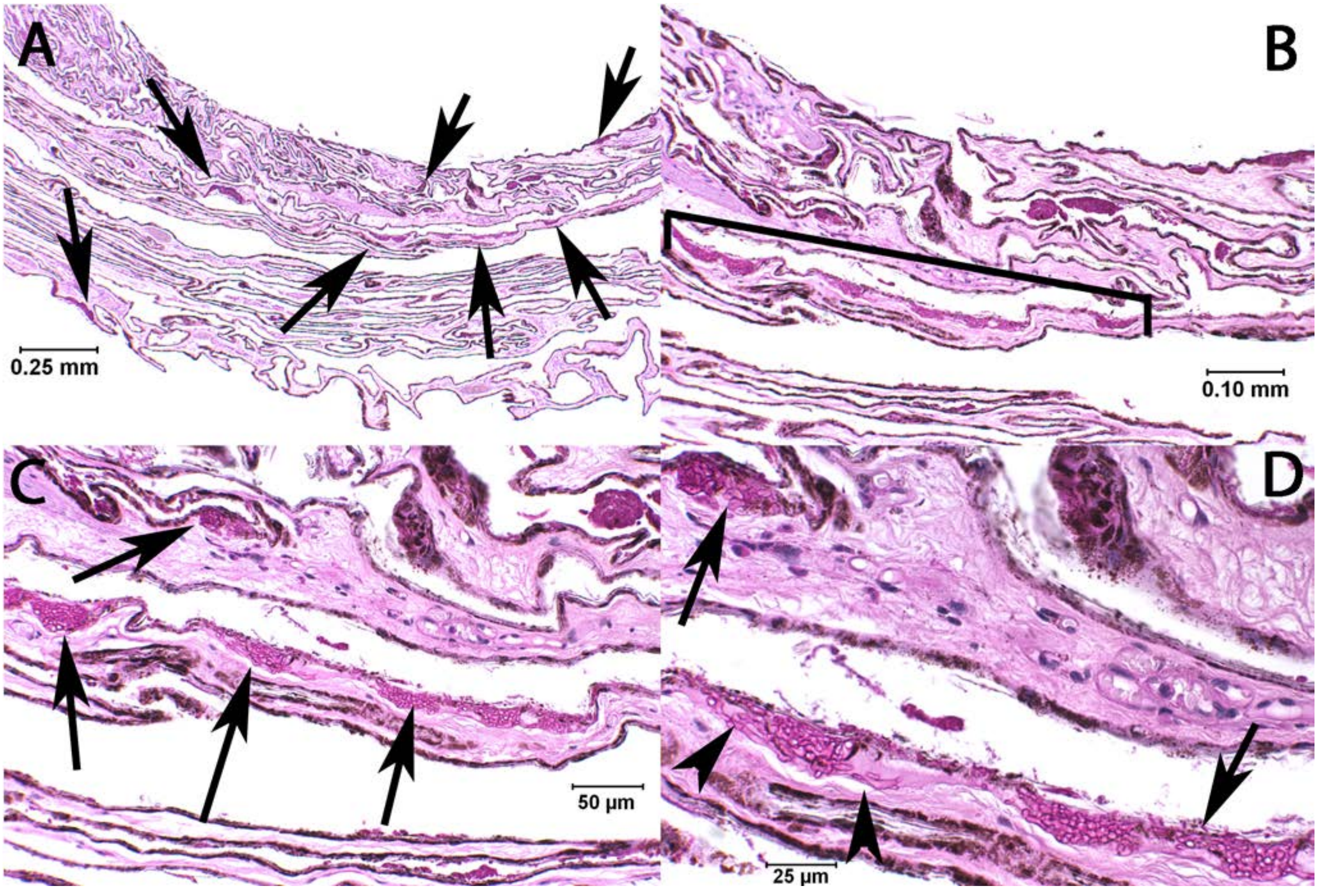

Appendix 2, Figure S3. Wing membrane damage severity score = 3 (SS3), moderately severe damage due to WNS. Photomicrographs of periodic acid Schiff-stained 4-μm sections of wing membrane prepared as described above. A portion of a single roll of wing membrane from a little brown bat (*Myotis lucifugus*) contains numerous cupping erosions; only a subset of these erosions is marked (arrows). The cupping erosions are expanding and coalescing (bracket). Individual fungal hyphae are beginning to move beyond the interface of the fungal aggregate and invade the deeper dermis (arrowheads, C). Four magnifications of this field of view (A, B, C, D) have calibrations bars embedded in the image.

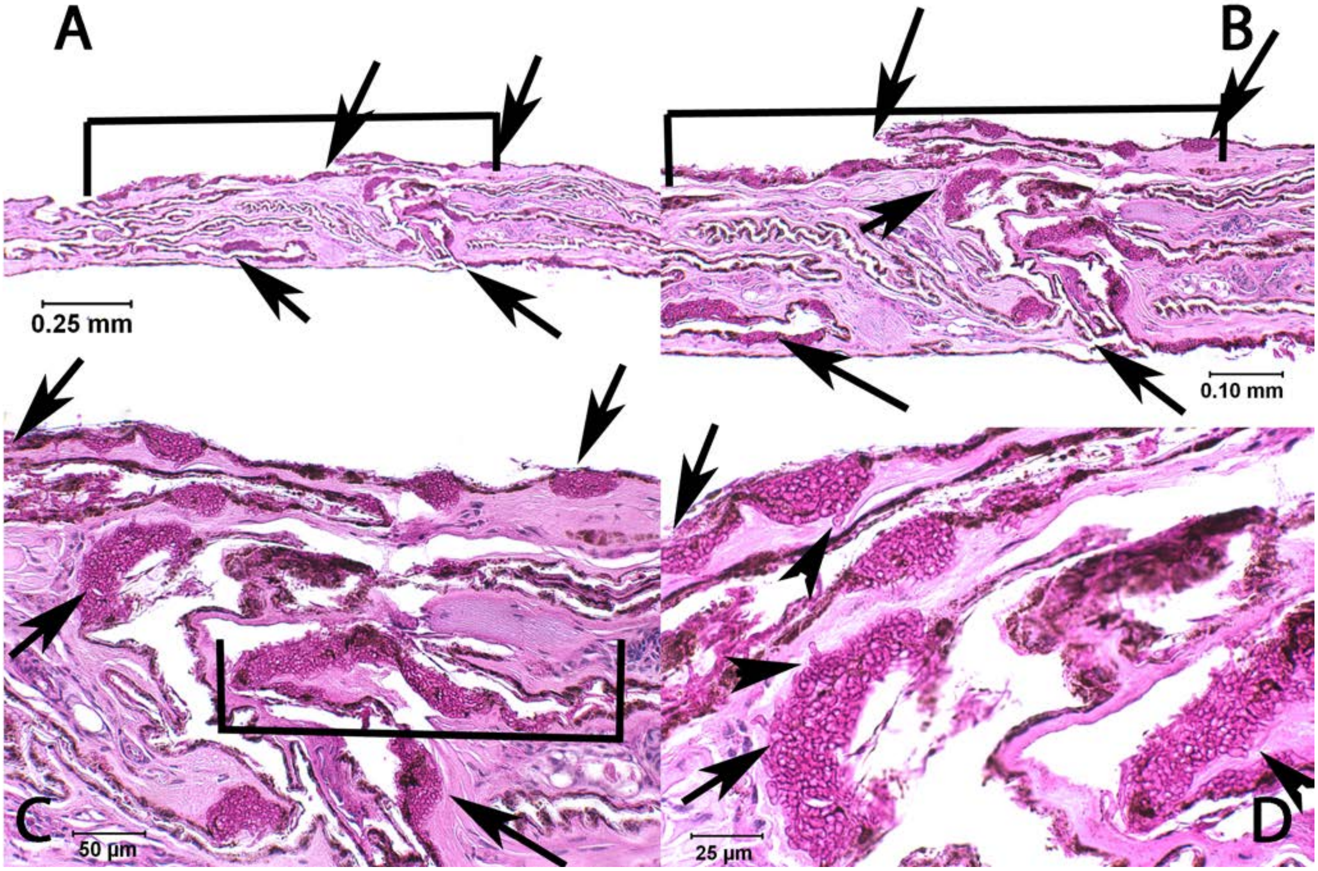

Appendix 2, Figure S4. Wing membrane damage severity score = 4 (SS4), severe damage due to WNS. Photomicrographs of periodic acid Schiff-stained 4-μm sections of wing membrane prepared as described above. A portion of a single roll of wing membrane from a little brown bat (*Myotis lucifugus*) containing more numerous and extensive erosions than Fig. S3, and many are approaching transmembrane invasion; only a subset of these erosions and ulcers are marked (arrows). Coalescing fungal aggregates (brackets) expand to cover more surface area of wing membrane. The morphology of the wing membrane becomes multifocally distorted in response to the extensive fungal invasion. Individual fungal hyphae are beginning to move beyond the interface of the fungal aggregate and invade the deeper dermis (arrowheads, C). Four magnifications of this field of view (A, B, C, D) have calibrations bars embedded in the image.

TABLE SUMMARIZING CRITERIA USED TO SCORE THE SEVERITY OF WNS-ASSOCIATED WING MEMBRANE DAMAGE

**Severity Score (SS) or Grade**	**Terminology**	**Number of wing membrane rolls out of the 6 with WNS cupping erosions**	**Number of WNS cupping erosions or ulcerations in the membrane rolls**
0	Not WNS	None	None
1	Mild	At least one	At least one erosion in any of the ‘rolls’
2	Moderate	At least 4/6	Approximately 10 erosions in each ‘roll’
3	Moderately severe	All affected 6/6	At least one ‘roll’ with more than 20
4	Severe	All affected 6/6	Most rolls with more than 20 erosions or ulcerations, some may have more than 100

References

S1. Meteyer CU, Buckles EL, Blehert DS, Hicks AC, Green DE, et al. (2009) Histopathologic criteria to confirm white-nose syndrome in bats. Journal of Veterinary Diagnostic Investigation 21: 411-414.

S2. Cryan PM, Meteyer CU, Boyles JG, Blehert DS (2010) Wing pathology of white-nose syndrome in bats suggests life-threatening disruption of physiology. BMC Biol 8: 135.
